# Cost-Effectiveness Analysis of Camrelizumab Plus Chemotherapy vs. Chemotherapy Alone as the First-Line Treatment in Patients With IIIB–IV Non-Squamous Non-Small Cell Lung Cancer (NSCLC) Without EGFR and ALK Alteration from a Perspective of Health - Care System in China

**DOI:** 10.3389/fphar.2021.735536

**Published:** 2021-12-24

**Authors:** Chen Zhu, Xiao-xuan Xing, Bin Wu, Gang Liang, Gang Han, Cai-xia Lin, Hong-mei Fang

**Affiliations:** ^1^ Department of Pharmacy, Sir Run Run Shaw Hospital, School of Medicine, Zhejiang University, Hangzhou, China; ^2^ Department of Pharmacy, Xuanwu Hospital of Capital Medical University, Beijing, China; ^3^ Medical Decision and Economic Group, Department of Pharmacy, Ren Ji Hospital, South Campus, School of Medicine, Shanghai Jiaotong University, Shanghai, China; ^4^ Department of Pharmacy, The People’s Hospital of Jiangshan, Jiangshan, China

**Keywords:** nonsquamous non-small cell lung cancer, NSCLC, camrelizumab, CAMEL, cost-effectiveness

## Abstract

**Objective:** The CAMEL clinical trial (412 patients were randomly assigned to either camrelizumab plus chemotherapy (n = 205) or chemotherapy alone (n = 207)) demonstrated that camrelizumab plus chemotherapy (CC) improved the overall survival time (OS) and progression-free survival time (PFS) of patients with metastatic nonsquamous non-small cell lung cancer (non-sq NSCLC) without epidermal growth factor receptor (EGFR) or anaplastic lymphoma kinase (ALK) mutations (EGFRm and ALKm) vs. chemotherapy (C) alone. Our objective was to conduct a cost-effectiveness analysis of CC vs. C from a perspective of health - care system in China with a lifetime horizon to identify whether it will be cost-effective.

**Materials and Methods:** A partitioned survival model (PSM) was applied for patients with IIIB–IV non-sq NSCLC without EGFRm and ALKm. Transition parameters and proportions of three health states were derived from the CAMEL trial. The model was designed using a lifetime horizon, a 21-day cycle, and a 5% discount rate of costs and outcomes. It was deemed cost-effective in China if the incremental cost-effectiveness ratio (ICER) value is less than $32,457 per quality adjusted life-year (QALY). Deterministic and probabilistic sensitivity analyses were performed to verify the influence of parameter uncertainty on the results.

**Results:** In the base-case analysis, we found that the ICER of CC compared with C is $-7,382.72/QALY which meant that CC had lower costs and better outcomes. The results of the sensitivity analyses demonstrated that the result was robust for the ICERs never transcending the willingness-to-pay (WTP) threshold.

**Conclusion:** Camrelizumab plus chemotherapy is an obviously cost-effective therapeutic regime for patients of IIIB–IV non-sq NSCLC without EGFRm and ALKm in China at a $32,457 WTP threshold.

## Introduction

Lung carcinoma is the most common malignancy all over the world. It is also the most frequent cause of cancer-related death in human beings, which is estimated to be accountable for nearly 1/5 deaths for cancer (Fitzmaurice et al., 2018). In 2015, the aggregate expenditure of lung cancer treatment reached ¥24.31 billion in China, representing 0.6% of the total health cost (Wan et al., 2020). Non-small cell lung cancer (NSCLC) bears approximately 85% of the whole lung cancer cases (Reck and Rabe, 2017), and what is worse, about more than 70% of patients have developed to the advanced stage at the time of diagnosis, leaving a 5-year survival rate of 18% (Herbst et al., 2018; Miller et al., 2019). Platinum-doublet chemotherapy (±bevacizumab) has remained the main first-line therapy for patients with metastatic NSCLC for a long time until immune checkpoint inhibitor (ICI) comes out (Sandler et al., 2006; Zhou et al., 2015; Planchard et al., 2018), such as inhibitors of programmed death 1 (PD-1) and its ligand PD-L1, which are effective therapies for metastatic NSCLC lacking sensitizing epidermal growth factor receptor (EGFR) or anaplastic lymphoma kinase (ALK) mutations (EGFRm or ALKm).

Several elegant clinical trials (Keynote-189, IMpower-130, IMpower-150, IMpower-132, and CAMEL) have shown that compared with chemotherapy alone, PD-1 or PD-L1 plus chemotherapy can significantly improve progression-free survival (PFS) and overall survival (OS) in patients with advanced non-squamous NSCLC (non-sq NSCLC), irrespective of the PD-L1 expression level (Gandhi et al., 2018; Socinski et al., 2018; West et al., 2019; Nishio et al., 2021; Zhou et al., 2021). Recently, a meta-analysis (Ferrara et al., 2020) shows that single-agent ICI in patients with NSCLC and PD-L1 ≥50% probably lead to a higher OS rate (hazard ratio (HR) 0.68, 95% confidence interval (CI) [0.60–0.76]) and may improve PFS (HR 0.68, 95% CI [0.52–0.88]) and overall response rate (ORR) (risk ratio (RR) 1.40, 95% CI [1.12–1.75]) when compared to platinum-based chemotherapy and may also lead to a lower rate of adverse events (AEs) (RR 0.41, 95% CI [0.33–0.50]) and higher health-related quality of life (HRQoL) (RR 1.51, 95% CI [1.08–2.10]). Combined ICI in patients with NSCLC and PD-L1 ≥50% also probably lead to a higher OS rate (HR 0.72, 95% CI [0.59–0.89]), but its effect on PFS, ORR, and HRQoL is unknown due to a lack of data. The rate of AEs may not differ between groups. The CAMEL study was the first phase three study for evaluating immunotherapy plus chemotherapy in Chinese patients. Camrelizumab (SHR-1210) is a humanized monoclonal antibody against PD-1, and the CAMEL trial demonstrated the remarkable clinical benefits of camrelizumab combined with chemotherapy in non-sq NSCLC as the first-line therapy (PFS 11.3 vs. 8.3 months, HR 0.60 [0.45–0.79], *p* = 0.0001; OS 27.9 vs. 20.5 months, HR 0.73 [0.55–0.96], *p* = 0.0117) (Zhou et al., 2021). Besides, in June 2020, the combination regimen of camrelizumab + standard chemotherapy (platinum and pemetrexed), as the first-line therapy for patients with metastatic non-sq NSCLC without EGFRm or ALKm, was approved by the National Medical Products Administration (NMPA) in China and was included in the Guidelines of Chinese Society of Clinical Oncology (CSCO) (Non-Small Cell Lung Cancer) (CSoCOC, 2020). Moreover, the therapeutic regime mentioned above has been included in 2020 national medical insurance catalogue with more than 85% reduction in the price of camrelizumab.

Despite these encouraging results, relative higher cost of the combination therapy (camrelizumab with chemotherapy) compared with chemotherapy alone urges us to attach importance to the pharmacoeconomic evaluation. Accordingly, our goal was to conduct a cost-effectiveness analysis of camrelizumab + chemotherapy (CC) vs. chemotherapy alone (C) in the first line treatment of patients with IIIB–IV non-sq NSCLC without EGFRm and ALKm from a perspective of health - care system in China following a lifetime horizon to confirm whether it will be cost-effective.

## Materials and Methods

### Model Structure

**FIGURE 1:**
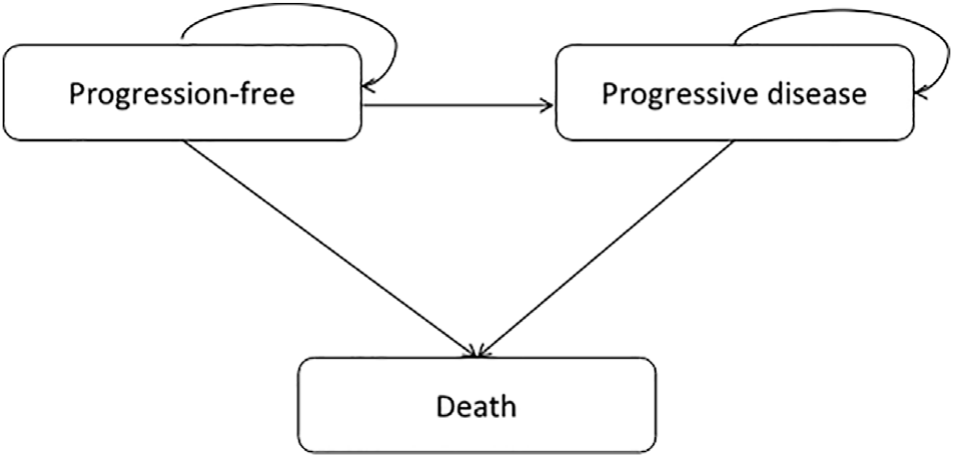
Structure of the partitioned-survival model.

A partitioned-survival model (PSM) was established depending on the clinical data from the phase three study (CAMEL) (Zhou et al., 2021) to estimate the costs and effectiveness of the two treatments, with three health states: PFS, progressive disease (PD), and death (Figure 1). The PSM structure eliminates the need to put forward assumptions for the transition of patients between different health states and enables the direct use of the trials’ Kaplan-Meier (K-M) curves to directly divide patients into different health states. Therefore, the estimation of patients’ proportion in each health state was acquired directly from the cumulative survival probabilities in OS and PFS curves by parametric functions fitting and extrapolation. The model compared the medical cost and health outcomes for two treatments: (C group) treated with chemotherapy (carboplatin and pemetrexed) vs. (CC group) treated with camrelizumab + chemotherapy combination (camrelizumab, carboplatin, and pemetrexed). Patients could receive subsequent treatment if the disease progressed or unacceptable AEs occurred. The population was a cohort with the same characteristics as those in the CAMEL trial (patients with IIIB–IV non-sq NSCLC without EGFRm and ALKm were aged 18–70 years [Median (IQR), 59 (54–64) years and 61 (53–65) years, respectively] and male accounted for 71 and 72%, respectively). The cycle length was 21-days in keeping with the treatment schedule reported by the CAMEL trial (Zhou et al., 2021) and the time horizon was the whole life so as to ensure there were less than 1% survivors. Costs, life years (LYs), quality-adjusted life years (QALYs), and incremental cost-effectiveness ratios (ICERs) were calculated in each treatment group. In line with Chinese pharmacoeconomic guidelines (Liu et al., 2020), both costs and benefits are discounted at 5% (range: 0–8%) per year. The initial state is assumed to be PFS, and death is the absorbing state.

### Efficacy Estimates

**FIGURE 2:**
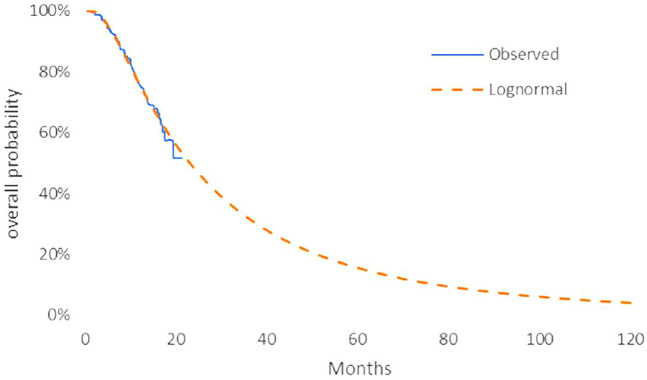
Parametric models for OS—camrelizumab plus chemotherapy. The log-normal model was chosen as the best fit model for the OS curve of the CC arm.

**FIGURE 3:**
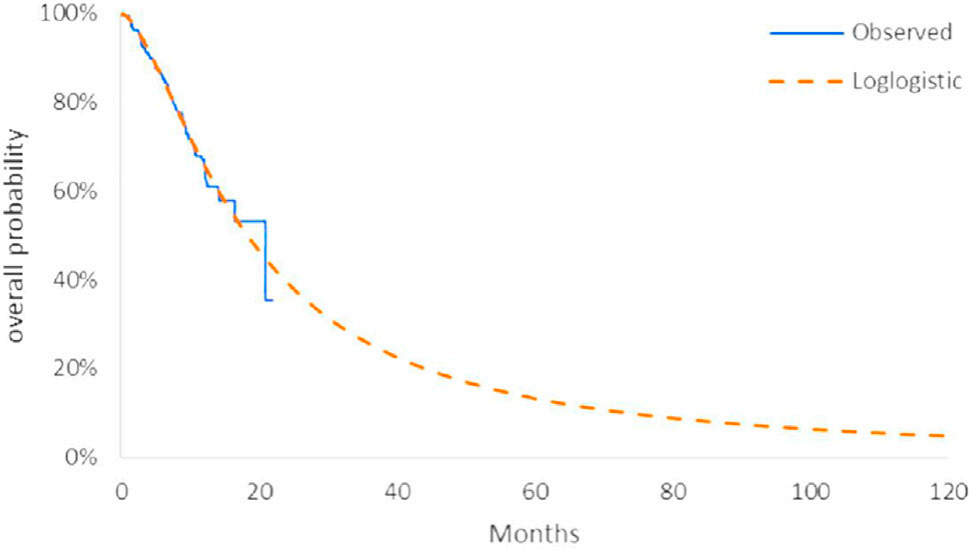
Parametric models for OS—chemotherapy. The log-logistic model was chosen as the best fit model for the OS curve of the C arm.

**FIGURE 4:**
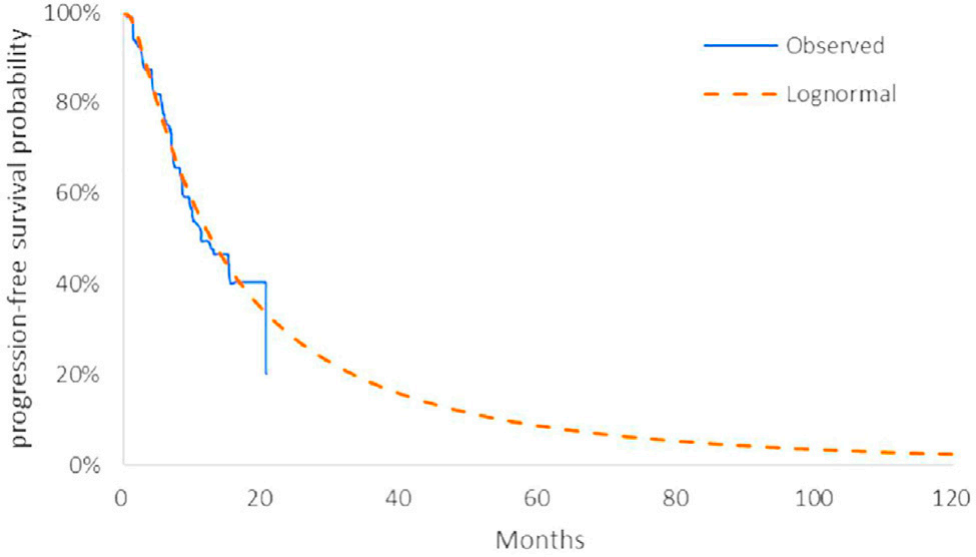
Parametric models for PFS—camrelizumab plus chemotherapy. The log-normal model was chosen as the best fit model for the PFS curve of the CC arm.

**FIGURE 5:**
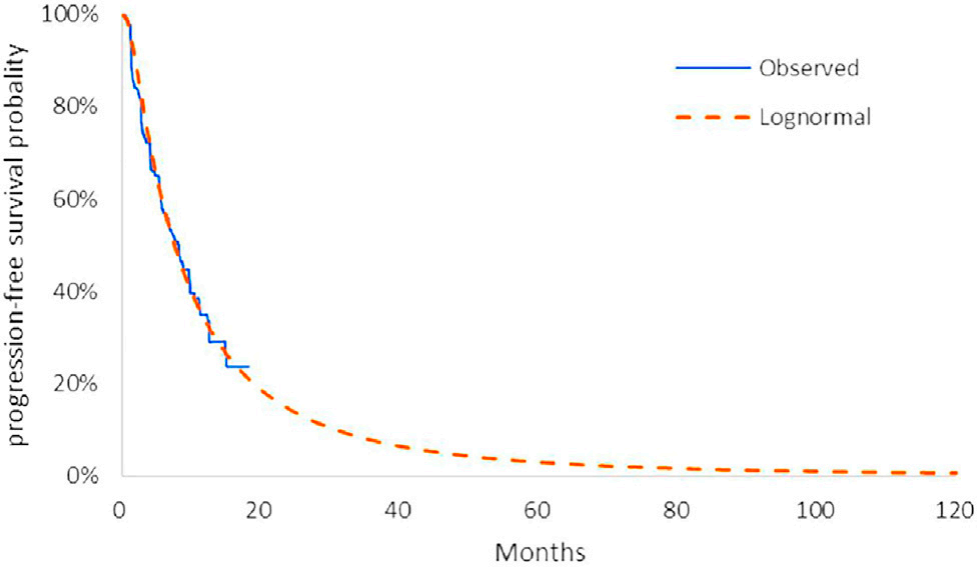
Parametric models for PFS—chemotherapy. The log-normal model was chosen as the best fit model for the PFS curve of the C arm.

The model transition parameters and proportions were directly obtained from the results of CAMEL (Zhou et al., 2021). We used the GetData Graph Digitizer (version 2.26) to collect the data points from the K–M curves (PFS and OS curves) of the two arms and followed the method of Guyot et al. (2012) to reconstruct estimates of underlying individual patient data (IPD) over the clinical trial time. In terms of the IPD out of the clinical trial time, standard parametric models were used for parametric extrapolation and long-term survival estimates by using the R version 4.1.0 (https://www.r-project.org). Specifically, six parametric functions were considered including exponential, Weibull, Gompertz, log-logistic, log-normal, and generalized gamma distributions. And then, a series of methods were applied to evaluate the goodness-of-fit of each parametric survival model under appropriate circumstances, such as visual inspection and Akaike information criterion (AIC)/Bayesian information criteria (BIC) tests proposed by NICE DSU technical support document 14 (TSD14) (Latimer, 2013) (table in appendix SM1-4). Lower AIC and BIC values indicate better fit of the selected model. Superimposed graphs of the K-M curves from the trial and the predicted curves based on the best fitting parametric survival models are presented in Figure 2, Figure 3, Figure 4, and Figure 5 to intuitively inspect the survival prediction.

The log-logistic model was chosen as the best fit model for the OS curve of C arm and log-normal model for CC arm and PFS curve of C arm. Considerations were as follows: (1) the lowest AIC and BIC values among all survival models; (2) the best fit with the observed curves based on visual inspection.

### Utility Estimates

The health utility score reflects the level of physical, mental, and social functioning associated with a disease correlative health state that varies from 0 to 1, with 0 representing the worst health state/death and 1 representing the best. Average health utilities of the PFS and the PD state were 0.804 (Nafees et al., 2017) and 0.321 (Nafees et al., 2017), respectively, which were obtained from the previously published literature (Nafees et al., 2017). The disutility values owing to 3/4 AEs were considered in our analysis (Nafees et al., 2008; Handorf et al., 2012), but only one-time assessment was carried out during the first cycle for simplification given the trivial influence of AE disutilities. The incidence of AEs was multiplied by the corresponding disutility value to assess the QALYs loss caused by AEs. In addition, ± 20% as the boundaries of the range were used in sensitivity analyses.

### Cost Inputs

In our study, only direct medical costs were considered, including cost of the drug utilization, PD-L1 test, main AEs, treatments for progression (including active treatments and supportive care), monitoring, and terminal care. Drug prices were estimated from the local bid-winning price (Drugdataexpy). Only severe AEs with great clinical impact, including anemia, neutropenia, and thrombocytopenia, were calculated because they had a relatively considerable influence on the economic evaluation by decreasing quality of life and increasing utilization of health resource. In addition, AE costs were calculated only once in the first cycle.

Costs of monitoring, AEs, terminal care, and PD-L1 tests were obtained from previously published studies (Wu et al., 2012; Zheng et al., 2018; Jiang and Wang, 2020; Wan et al., 2020). All patients were assumed to incur one-time PD-L1 test costs in the first cycle and one-time terminal care costs before death. Additionally, costs were discounted at an annual rate of 5% (Sanders et al., 2016). All costs were converted into United States dollars (USD) by exchange rate: 1 USD = 6.47 CYN. All these data are listed in Table 1. If the ICER is below $32,457 threshold (three times GDP per capita of China in 2020, ¥210,000.00), the treatment is generally considered to be cost-effective.

**TABLE 1:** Costs and health utilities inputs

Variable	Base-line value	Range		References	Distribution
		Minimum	Maximum		
Cycle	21d				
Horizon	Lifetime				
Willingness-to-pay (WTP) threshold, $/QALY	32,457				
Discount rate	0.05	0	0.08		
Utility					
Utility - PFS in first-line treatment	0.804	0.64	0.96	(Nafees et al., 2017)	Beta
Utility - PD	0.321	0.26	0.39		Beta
Disutility due to AEs					
Neutropenia	-0.2	-0.16	-0.24	(Nafees et al., 2017)	Beta
Anaemia	-0.07	-0.056	-0.084	(Nafees et al., 2017) Assumed equal to fatigue	Beta
Thrombocytopenia	-0.2	-0.16	-0.24	Assumed equal to neutropenia	Beta
Total disutility due to AEs (only one-time during the first cycle for simplification given the trivial impact of AE disutilities)					
CC group	-0.123	-0.099	-0.148		Beta
C group	-0.092	-0.073	-0.110		Beta
Risk for main adverse events in CC group					
Neutropenia	0.38	0.30	0.46	CAMEL trial (Zhou et al., 2021)	Beta
Anaemia	0.19	0.15	0.23		Beta
Thrombocytopenia	0.17	0.14	0.20		Beta
Risk for main adverse events in C group					
Neutropenia	0.30	0.24	0.36	CAMEL trial (Zhou et al., 2021)	Beta
Anaemia	0.11	0.09	0.13		Beta
Thrombocytopenia	0.12	0.1	0.14		Beta
Costs of main AEs, $/event					
Neutropenia	466	415	508	(Wu et al., 2012)	Gamma
Anaemia	537	478	585		Gamma
Thrombocytopenia	6397	5117	7676	(Zheng et al., 2018)	Gamma
Total costs of main AEs, $ (only one-time during the first cycle for simplification given the trivial impact of AE costs)					
CC group	1366.600	1093.280	1639.920		Gamma
C group	966.510	773.208	1159.812		Gamma
Drug cost, $/mg					
Camrelizumab	2.26	1.81	2.71	Local charge	Gamma
Pemetrexed	0.86	0.69	1.03		Gamma
Carboplatin	0.16	0.13	0.19		Gamma
Nivolumab	14.30	11.44	17.16		Gamma
Docetaxel	3.00	2.40	3.60		Gamma
Drug cost, $/cycle					
CC group (initial treatment, for 5 cycles followed by camrelizumab 200 mg + pemetrexed 500 mg/m^2^, q3w maintenance)					
Camrelizumab	452.00	361.60	542.40	Local charge	Gamma
Pemetrexed	740.00	592.00	888.00		Gamma
Carboplatin	87.50	70.00	105.00		Gamma
C group (initial treatment, for 5 cycles followed by pemetrexed 500 mg/m^2^, q3w maintenance)					
Pemetrexed	740.00	592.00	888.00	Local charge	Gamma
Carboplatin	87.50	70.00	105.00		Gamma
CC group subsequent therapy cost, $/cycle					
Docetaxel	405.00	324.00	486.00	Local charge	Gamma
Docetaxel_proportion	81.50%	65.20%	97.80%	CAMEL trial (Zhou et al., 2021)	Beta
Supportive care	338	159	476	(Lu et al., 2017)	Gamma
Supportive care_proportion	18.50%	14.80%	22.20%	CAMEL trial (Zhou et al., 2021)	Beta
Total_subsequent therapy cost, $/cycle	392.61	314.08	471.13		Gamma
C group subsequent therapy cost, $/cycle					
Docetaxel	405.00	324.00	486.00	Local charge	Gamma
Docetaxel_proportion	25.20%	20.16%	30.24%	CAMEL trial (Zhou et al., 2021)	Beta
Nivolumab	4182.75	3346.20	5019.30	Local charge	Gamma
Nivolumab_proportion	7.10%	5.68%	8.52%	CAMEL trial (Zhou et al., 2021)	Beta
Camrelizumab	452.00	361.60	542.40	Local charge	Gamma
Camrelizumab_proportion	62.20%	49.76%	74.64%	CAMEL trial (Zhou et al., 2021)	Beta
Supportive care	338	159	476	(Lu et al., 2017)	Gamma
Supportive care_proportion	5.50%	4.40%	6.60%	CAMEL trial (Zhou et al., 2021)	Beta
Total_subsequent therapy cost, $/cycle	698.77	559.02	838.52		Gamma
Monitoring costs, $/cycle	102.50	82.00	123.00	(Wan et al., 2020)	Gamma
Cost for PD-L1 test (one-time cost in the first cycle), $	48.50	38.80	58.20		Gamma
Terminal care (one-time cost), $	2464.50	1971.60	2957.40	0 (Jiang and Wang, 2020)	Gamma

PD-L1, programmed death ligand 1; CC, group, camrelizumab plus chemotherapy group; C group, chemotherapy group.

### Clinical Inputs

**FIGURE 6:**
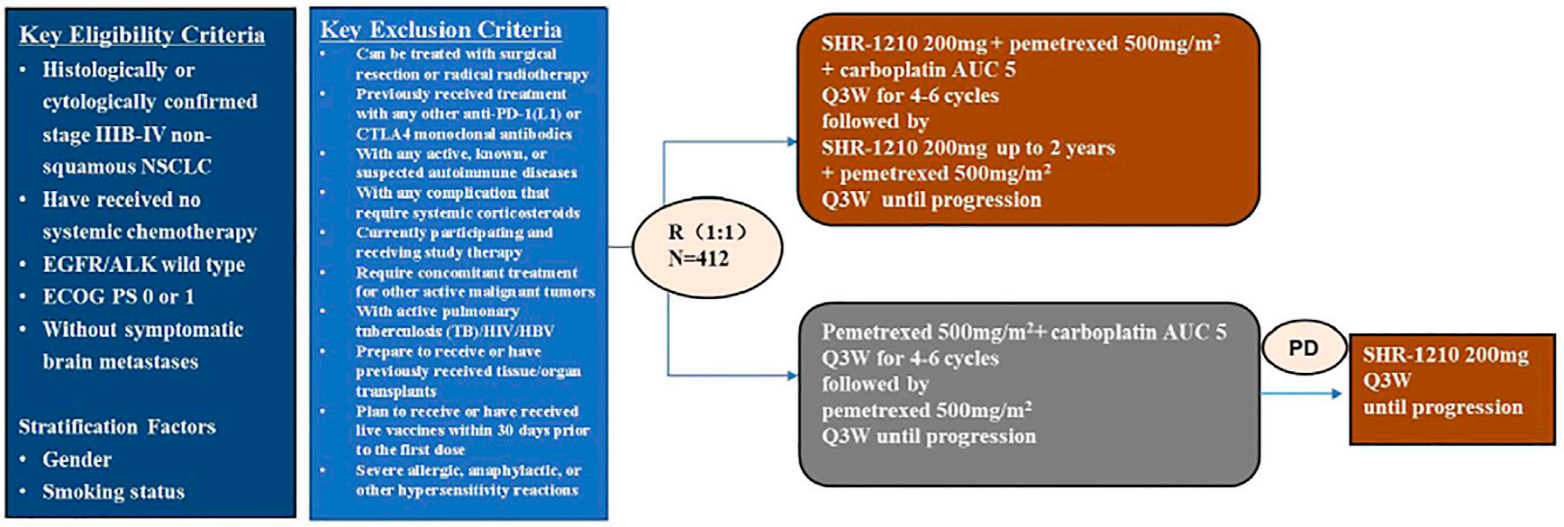
Flowchart of treatment regime along with inclusion and exclusion criteria. Reference: CAMEL (NCT03134872) Protocol No.: SHR-1210-III-303-NSCLC.

According to the phase three CAMEL trial (Figure 6), the patients in the CC group (N = 205) received camrelizumab 200 mg and pemetrexed 500 mg/m^2^ and carboplatin at an area under the curve (AUC) 5 mg/ml per min, administered as intravenous infusion on Day 1 of each 21-day cycle for 4-6 cycles followed by optional camrelizumab 200 mg and pemetrexed 500 mg/m^2^ every 3 weeks (Q3W) maintenance for the remainder of the study or until recorded PD, intolerable AEs, death, or study completion; the patients in the C group (N = 207) received pemetrexed + platinum on Day 1 per 21-day cycle for 4-6 cycles followed by pemetrexed 500 mg/m^2^ Q3W maintenance. Patients in the C group who were confirmed disease development had an optional crossover to camrelizumab monotherapy according to investigators’ judgment, which was also taken into account in our PSM. The total exposure of camrelizumab should not be more than 2 years. After the initial treatment, 75 (81.5%, PD 92) in the CC group and 120 (94.5%, PD 127) patients in the C group received at least one subsequent anti-tumor therapy, and we assumed that the others (17 (18.5%) and 7 (5.5%), respectively) were treated with supportive care. In the C group, 79 (62.2%) patients transformed to camrelizumab monotherapy and 9 (7.1%) received other anti-PD-1 drugs, alone or combined with other therapies. Subsequent anticancer therapies included immunotherapy and chemotherapy. Referring to the other studies, we assumed that docetaxel was used as the standard second-line chemotherapy, and nivolumab and camrelizumab as the immunotherapy, which is commonly used in China. For dosage calculation, values of body surface area (BSA), weight, and creatinine clearance rate (Ccr) were 1.80 m^2^, 65kg, and 90 ml/min/1.73 m^2^, respectively (Wu et al., 2011).

(Costs and health utilities inputs were summarized in Table 1)

### Sensitivity Analysis

A series of sensitivity analyses were conducted to test the robustness of the model. One-way deterministic sensitivity analyses (DSA) were used to evaluate the impact of uncertainty of a single input variable on the ICER. All parameters were adjusted within the reported 95% confidence intervals (CI) or assuming reasonable ranges of the base case values (±20%) if 95% CIs were unavailable, in accordance with established approach (Zhang et al., 2012). In the probabilistic sensitivity analysis (PSA), a Monte Carlo simulation of 1000 iterations was generated by simultaneously sampling the key model parameters from the pre-specified distributions. The variables about risk of main AEs, proportion of patients and utilities were assigned beta distributions, and the variables about costs used gamma distributions. Based on the data from 1000 iterations, a cost-effectiveness acceptability curve (CEAC) was created to represent the likelihood that the CC regime would be considered cost-effective compared with the C regime on the basis of a willingness to pay (WTP) threshold of $32,457 per QALY in China.

## Results

### Base Case Results

Over a lifetime horizon, the total QALYs for CC and C were estimated to be 1.55 and 1.16, respectively, and the PSM assessed the total costs to be $19,023.42 and $21,922.27, respectively, during that period, resulting in an ICER of $-7382.72/QALY (Table 2).

**TABLE 2:** Base-case results

		Cost ($)	LYs	QALYs
Treatments	Camrelizumab plus chemotherapy	19,023.42	2.68	1.55
	Chemotherapy	21,922.27	2.40	1.16
		Incremental cost	Incremental LYs	Incremental QALYs
Incremental changes	Difference	-2,898.85	0.28	0.39
	Incremental cost per LY gained		-10,353.04	
	Incremental cost per QALY gained		-7,382.72	

### Sensitivity Analysis

#### Deterministic Sensitivity Analysis Results

**FIGURE 7:**
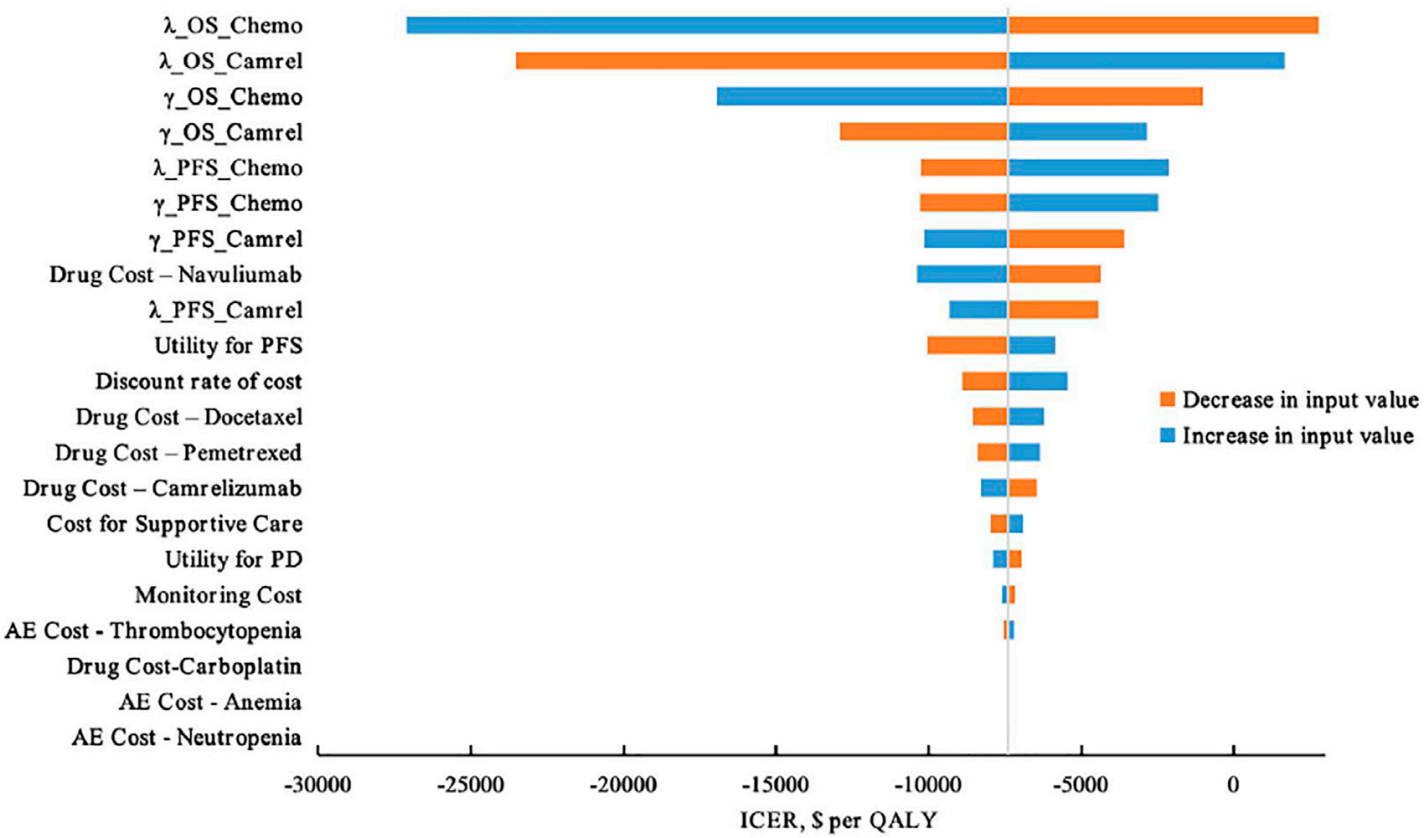
Top 20 DSA results ranked by impact on ICER values.

Tornado diagrams of one-way sensitivity analyses are shown in Figure 7. The results indicated that the parametric models for OS curves of both chemotherapy alone and combination arms were the most sensitive parameters in the model, which had the most significant impact on the ICER. Regardless of the variation of each parameter across the wide ranges, the ICER for the CC group compared with the C group remained less than $32,457/QALY (3×GDP per capita in China 2020), even less than $10,819 per QALY (1×GDP per capita in China 2020). The DSA results indicated that model results were robust.

#### Probabilistic Sensitivity Analysis Results

**FIGURE 8:**
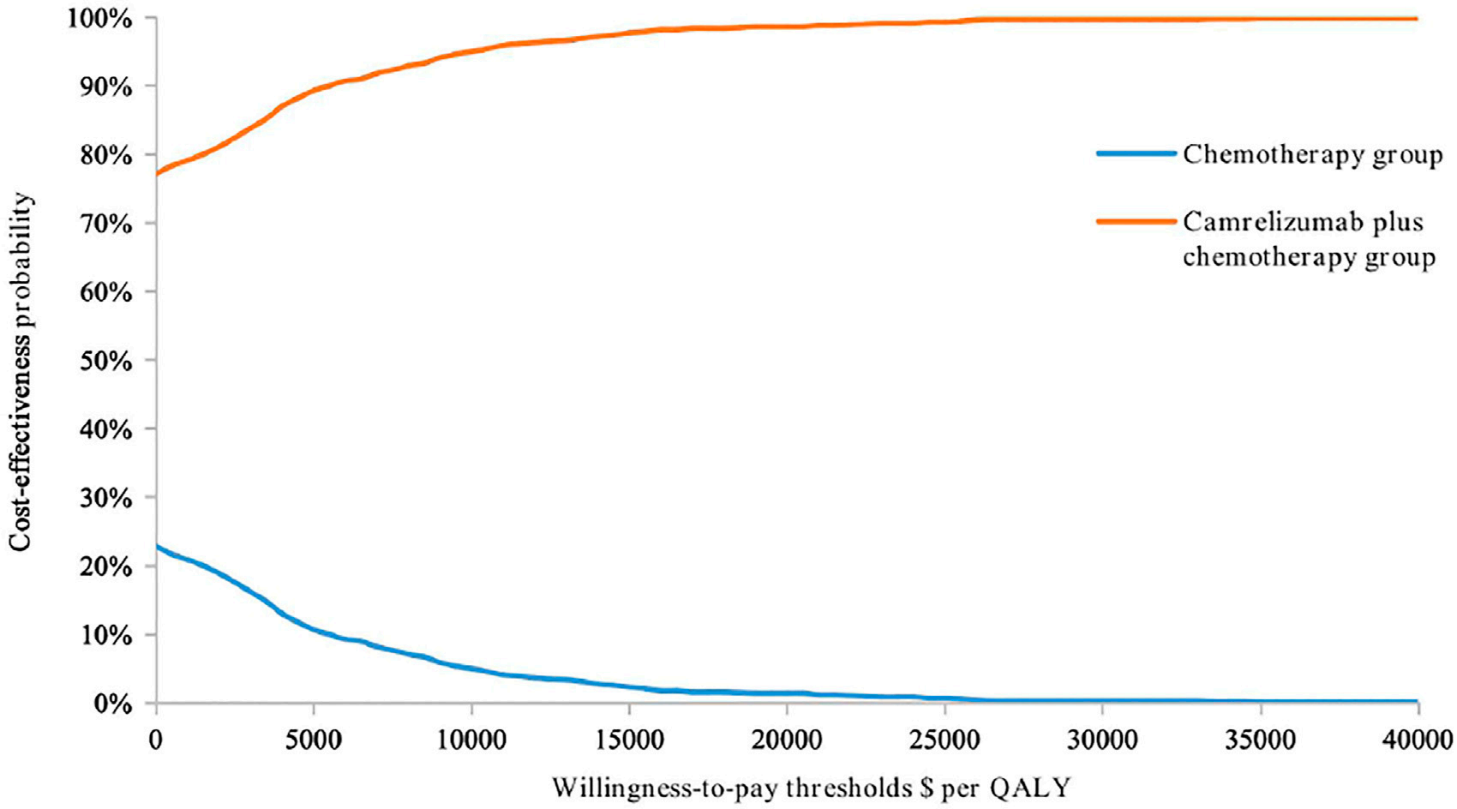
Cost-effectiveness acceptability curves. The cost-effectiveness acceptability Frontier shows the probability of strategies being cost-effective in two strategies. Compared with chemotherapy alone, the probability of camrelizumab plus chemotherapy being cost-effective at the specified WTP threshold of $32,457 per QALY gained is nearly 100%.

**FIGURE 9:**
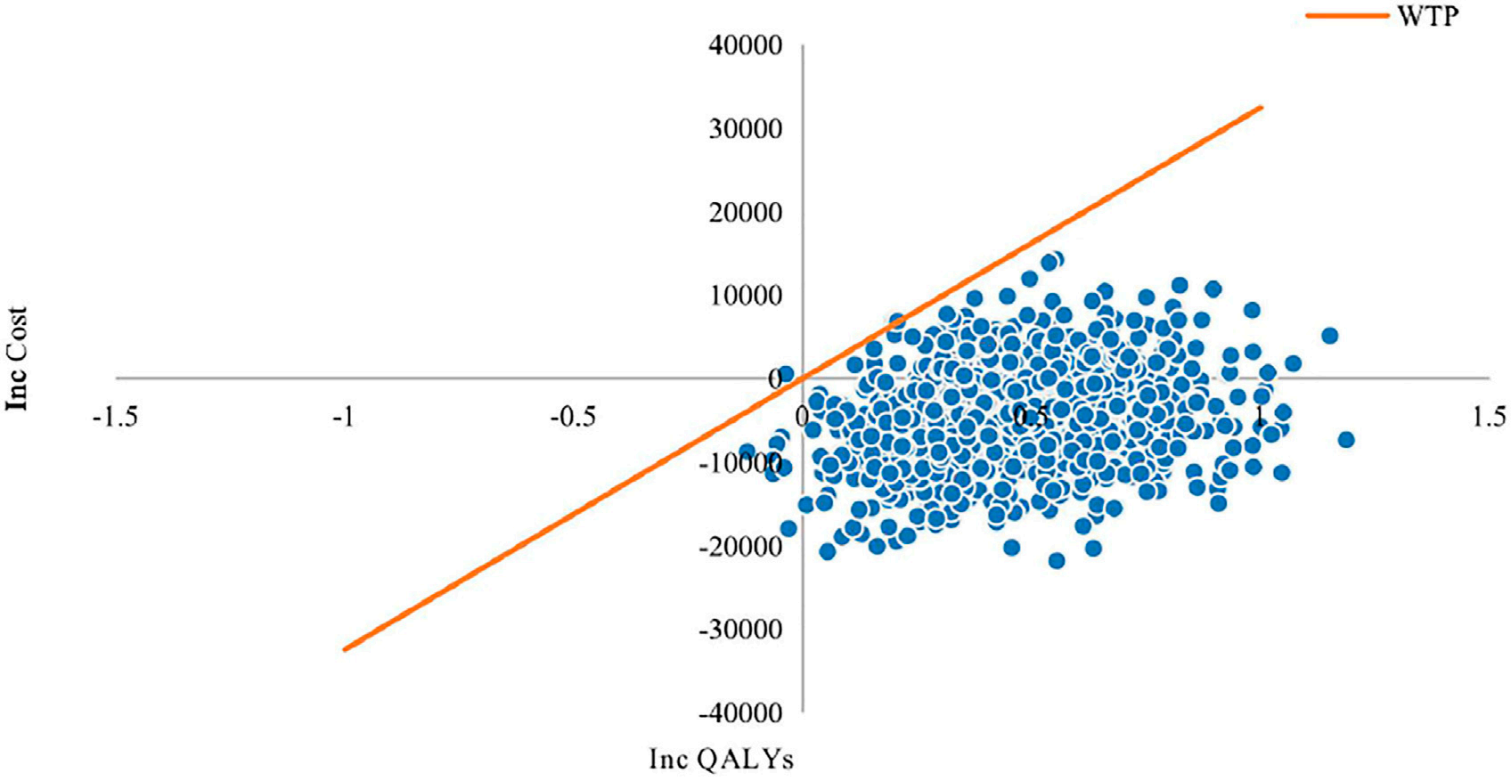
Cost-effectiveness plane with scatter plot of incremental costs and incremental QALYs (WTP = $32,457). The results of Monte Carlo simulation of 1000 iterations show that in most cases camrelizumab and chemotherapy combination therapy generated more QALYs and less costs than chemotherapy alone.

Cost-effectiveness probabilistic acceptability curves (CEACs) (Figure 8) showed that compared with chemotherapy alone the probability of camrelizumab plus chemotherapy being cost-effective at the specified WTP threshold of $32,457 per QALY gained was 100%. The scatter plots shown in Figure 9 depict the results of the 1000 simulations of the probabilistic sensitivity analysis, in which most results generated more QALYs and less costs than chemotherapy alone.

## Discussion

### Summary and Interpretation of Results

To our knowledge, this evaluation was the first study to evaluate the cost-effectiveness of camrelizumab and chemotherapy combination therapy for the target patients with metastatic non-sq NSCLC without EGFRm or ALKm in China. Under the circumstance of the decreased price of camrelizumab from $3060.3 to $452.6, our results showed that CC strategy produced an extra 0.39 QALYs while saving $2,898.85, which resulted in an ICER of $-7,382.72/QALY. These findings suggested that the CC strategy was absolutely cost-effective in China, which was also proved by the CEACs (Figure 8). Recently, some scholars (Kazibwe et al., 2021) pointed out that using 1 to 3× GDP per capita as a cost-effectiveness threshold had resulted in a higher proportion of study interventions being found as cost-effective, which might lead to a loss of would-be health gains from deployed resources for health. However, in our study, even if the opportunity cost threshold (or 0.5× GDP per capita) is used, the CC scheme is nearly 90% likely to be cost-effective, which means that our results are robust.

Compared with other PD1/PD-L1-chemotherapy combination therapeutic regimes, including pembrolizumab, atezolizumab, and sindilimab, which were approved in China or abroad as the first line therapy for patients with metastatic non-sq NSCLC without EGFRm or ALKm, only camrelizumab-chemotherapy combination therapeutic regime showed cost-effectiveness in China according to current research. For pembrolizumab, Jiang et al. (2020), Wan et al. (2020) and Wu et al. (2020) had developed studies to assess the cost-effectiveness of combination therapy by partitional survival model, Markov model, and decision tree plus Markov model, respectively. They found that the ICERs of combination treatment vs. chemotherapy alone were $96,644/QALY, $92,533/QALY and higher than $40,000/QALY, respectively, which highly exceed the WTP threshold in China (3 times of GDP per capita). So that, compared with chemotherapy alone, the combination treatments showed no cost-effectiveness. In terms of atezolizumab, Yang et al. (2021) found that the probability of atezolizumab plus chemotherapy (ICER $325,328.71/QALY) was cost-effective was 0% at a WTP value of $30,828/QALY (Table3). Based on these findings, our study may be useful for national medical insurance negotiation and decision-making.

**TABLE 3:** Summary of cost-effectiveness analyses in China for other PD1/PD-L1-chemotherapy combination therapeutic regimes in advanced non-squamous NSCLC

Study	Country	Disease	Model	WTP threshold	ICER
Pembrolizumab + Chemotherapy vs. Chemotherapy					
(Jiang and Wang, 2020)	China	Metastatic non-squamous NSCLC	Partitional survival model	_	$96 644/QALY
(Wan et al., 2020)		Non-squamous NSCLC	Markov model	$27 351/QALY	$92 533/QALY
(Wu and Lu, 2020)		Metastatic NSCLC	Decision tree and Markov model	$29 196/QALY	Higher than $40,000/QALY
Atezolizumab + Chemotherapy vs Chemotherapy					
(Yang et al., 2021)	China	Advanced non-squamous NSCLC	Markov model	$30 828/QALY	$325,328.71/QALY
Sindilimab + Chemotherapy vs. Chemotherapy (not found)					

### Strengths and Limitations

A major strength of the basic analysis was its dependence on a direct comparison of CC and C alone, using data and information from a randomized controlled trial. The second strength was that the population included in the trial and analysis were Chinese, which eliminated the influence of different races. Besides, the PSM structure was unnecessary to produce assumptions for the transition of patients between health states but enabled researchers to partition patients to different health states directly based on the trials’ K-M curves.

Analysis limitations were as follows: First, for the longer-term extrapolation of PFS and OS, there existed an inherent uncertainty. Besides the choice of follow-up treatment would be different according to the individual situation of patients, for which additional data from real world studies could help in validating the model over the long-term. Second, the values of utilities and disutilities were from published studies, some of which were not based on Chinese populations, and the differences caused by different treatment methods were not distinguished. Nafees et al. (2017) figured out that the values of utilities for NSCLC in China were higher than other countries. Third, the published data of the phase three CAMEL trial was the interim results with a median follow-up duration of 11.9 months (IQR 9.0–14.9). Thus, it might be possible that the prediction of cost-effectiveness could be changed with additional follow-up. In addition, this study was a secondary analysis of data from the published literature which might limit the conclusions.

## Conclusion

From a perspective of health - care system in China, the current model predicted that camrelizumab-chemotherapy combination therapeutic regime would offer obviously marked benefits to patients of IIIB–IV non-sq NSCLC without EGFR and ALK alteration in comparison with chemotherapy alone.

## Data Availability

The original contributions presented in the study are included in the article/Supplementary Material. Further inquiries can be directed to the corresponding authors.

## Appendix A1:

**TABLE A1:** Summary of goodness of fit statistics for camrelizumab + chemotherapy combination - OS

Treatment	Efficacy inputs	Parametric function	AIC	BIC
Camrelizumab + Chemotherapy	OS	Exponential	334.0987	337.4217
		Weibull	315.6491	322.2951
		Log-logistic	315.0087	321.6547
		**Log-normal**	**314.8133**	**321.4593**
		Gompertz	321.4039	328.0499
		Generalized gamma	316.5062	326.4752

Abbreviations: OS, overall survival; AIC, Akaike information criterion; BIC, Bayesian information criterion

**TABLE A2:** Summary of goodness of fit statistics for camrelizumab + chemotherapy combination - PFS

Treatment	Efficacy inputs	Parametric function	AIC	BIC
Camrelizumab + Chemotherapy	PFS	Exponential	431.5503	434.8733
		Weibull	426.7328	433.3788
		Log-logistic	422.5138	429.1598
		**Log-normal**	**421.8761**	**428.5221**
		Gompertz	432.1431	438.7892
		Generalized gamma	423.8371	433.8061

Abbreviations: PFS, progression-free survival; AIC, Akaike information criterion; BIC, Bayesian information criterion

**TABLE A3:** Summary of goodness of fit statistics for chemotherapy - OS

Treatment	Efficacy inputs	Parametric function	AIC	BIC
Chemotherapy	OS	Exponential	402.3452	405.6779
		Weibull	395.5862	402.2517
		**Log-logistic**	**394.6359**	**401.3014**
		Log-normal	395.8036	402.4691
		Gompertz	399.5324	406.1979
		Generalized gamma	396.5906	406.5887

Abbreviations: OS, overall survival; AIC, Akaike information criterion; BIC, Bayesian information criterion

**TABLE A4:** Summary of goodness of fit statistics for chemotherapy - PFS

Treatment	Efficacy inputs	Parametric function	AIC	BIC
Chemotherapy	PFS	Exponential	476.3160	479.6487
		Weibull	473.2939	479.9594
		Log-logistic	467.4051	474.0705
		**Log-normal**	**460.5066**	**467.1720**
		Gompertz	477.7013	484.3667
		Generalized gamma	458.8144	468.8125

Abbreviations: PFS, progression-free survival; AIC, Akaike information criterion; BIC, Bayesian information criterion
