# Supplementary material for: Cost-Effectiveness Analysis of Camrelizumab Plus Chemotherapy vs. Chemotherapy Alone as the First-Line Treatment in Patients With IIIB–IV Non-Squamous Non-Small Cell Lung Cancer (NSCLC) Without EGFR and ALK Alteration from a Perspective of Health - Care System in China
Source: Front Pharmacol. 2021 Dec 24;12:735536. doi: 10.3389/fphar.2021.735536 (PMC8740086; doi:10.3389/fphar.2021.735536)
Supplement: Supplementary file 1 [file Table1.DOCX]

**Appendix (Supplementary materials)**

Tab. SM1. Summary of goodness of fit statistics for camrelizumab + chemotherapy combination - OS

| Treatment | Efficacy inputs | Parametric Function | AIC | BIC |
| --- | --- | --- | --- | --- |
| Camrelizumab  +  Chemotherapy | OS | Exponential | 334.0987 | 337.4217 |
|  |  | Weibull | 315.6491 | 322.2951 |
|  |  | Log-logistic | 315.0087 | 321.6547 |
|  |  | **Log-normal** | **314.8133** | **321.4593** |
|  |  | Gompertz | 321.4039 | 328.0499 |
|  |  | Generalized gamma | 316.5062 | 326.4752 |
| Abbreviations: OS, overall survival; AIC, Akaike information criterion; BIC, Bayesian information criterion | | | | |

Tab. SM2. Summary of goodness of fit statistics for camrelizumab + chemotherapy combination - PFS

| Treatment | Efficacy inputs | Parametric Function | AIC | BIC |
| --- | --- | --- | --- | --- |
| Camrelizumab  +  Chemotherapy | PFS | Exponential | 431.5503 | 434.8733 |
|  |  | Weibull | 426.7328 | 433.3788 |
|  |  | Log-logistic | 422.5138 | 429.1598 |
|  |  | **Log-normal** | **421.8761** | **428.5221** |
|  |  | Gompertz | 432.1431 | 438.7892 |
|  |  | Generalized gamma | 423.8371 | 433.8061 |
| Abbreviations: PFS, progression-free survival; AIC, Akaike information criterion; BIC, Bayesian information criterion | | | | |

Tab. SM3. Summary of goodness of fit statistics for chemotherapy - OS

| Treatment | Efficacy inputs | Parametric Function | AIC | BIC |
| --- | --- | --- | --- | --- |
| Chemotherapy | OS | Exponential | 402.3452 | 405.6779 |
|  |  | Weibull | 395.5862 | 402.2517 |
|  |  | **Log-logistic** | **394.6359** | **401.3014** |
|  |  | Log-normal | 395.8036 | 402.4691 |
|  |  | Gompertz | 399.5324 | 406.1979 |
|  |  | Generalized gamma | 396.5906 | 406.5887 |
| Abbreviations: OS, overall survival; AIC, Akaike information criterion; BIC, Bayesian information criterion | | | | |

Tab. SM4. Summary of goodness of fit statistics for chemotherapy - PFS

| Treatment | Efficacy inputs | Parametric Function | AIC | BIC |
| --- | --- | --- | --- | --- |
| Chemotherapy | PFS | Exponential | 476.3160 | 479.6487 |
|  |  | Weibull | 473.2939 | 479.9594 |
|  |  | Log-logistic | 467.4051 | 474.0705 |
|  |  | **Log-normal** | **460.5066** | **467.1720** |
|  |  | Gompertz | 477.7013 | 484.3667 |
|  |  | Generalized gamma | 458.8144 | 468.8125 |
| Abbreviations: PFS, progression-free survival; AIC, Akaike information criterion; BIC, Bayesian information criterion | | | | |
